# Supplementary material for: Polycystic Ovary Syndrome Susceptibility Loci Inform Disease Etiological Heterogeneity
Source: J Clin Med. 2021 Jun 18;10(12):2688. doi: 10.3390/jcm10122688 (PMC8234947; doi:10.3390/jcm10122688)
Supplement: Supplementary file 1 [file jcm-10-02688-s001.zip › SupplementaryFigureS1.pdf]

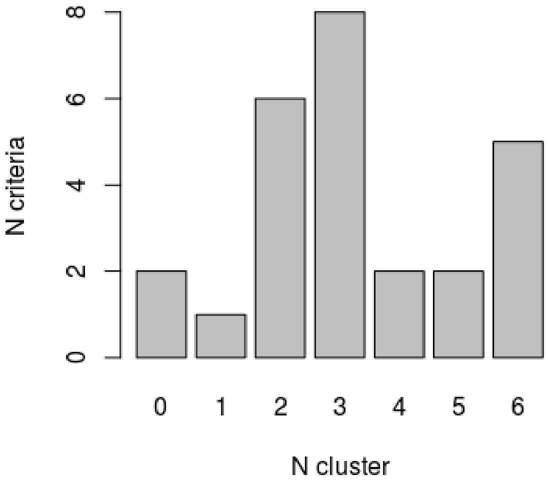

Figure S1. Using NbClust to identify the best number of clusters for k-means clustering. X-axis represents the number of clusters; y-axis represents of the number of criteria to support the number of clusters by NbClust package.
